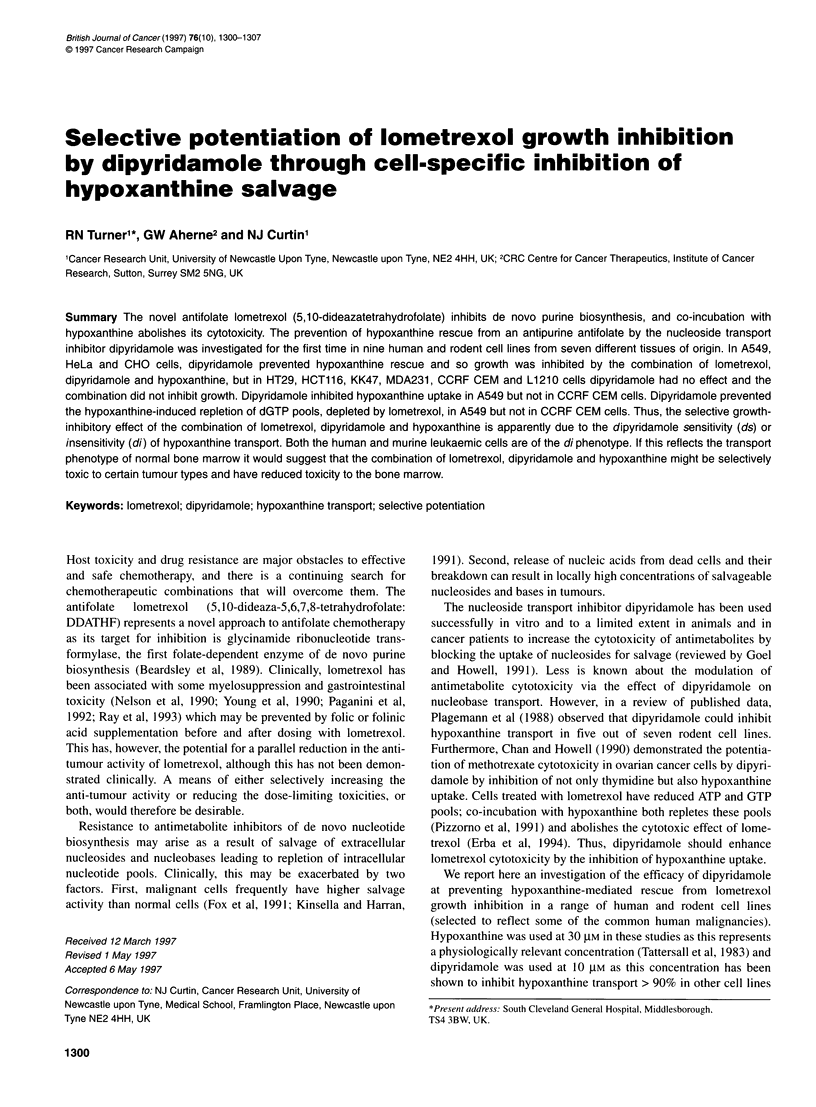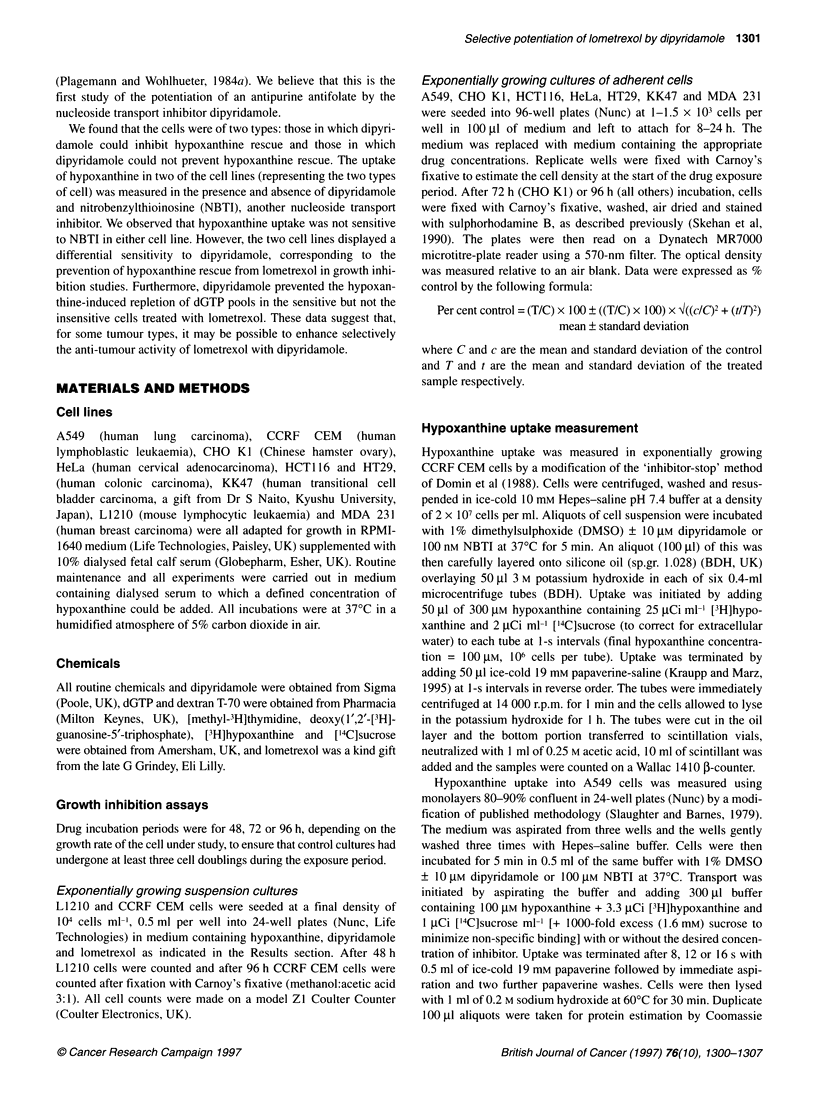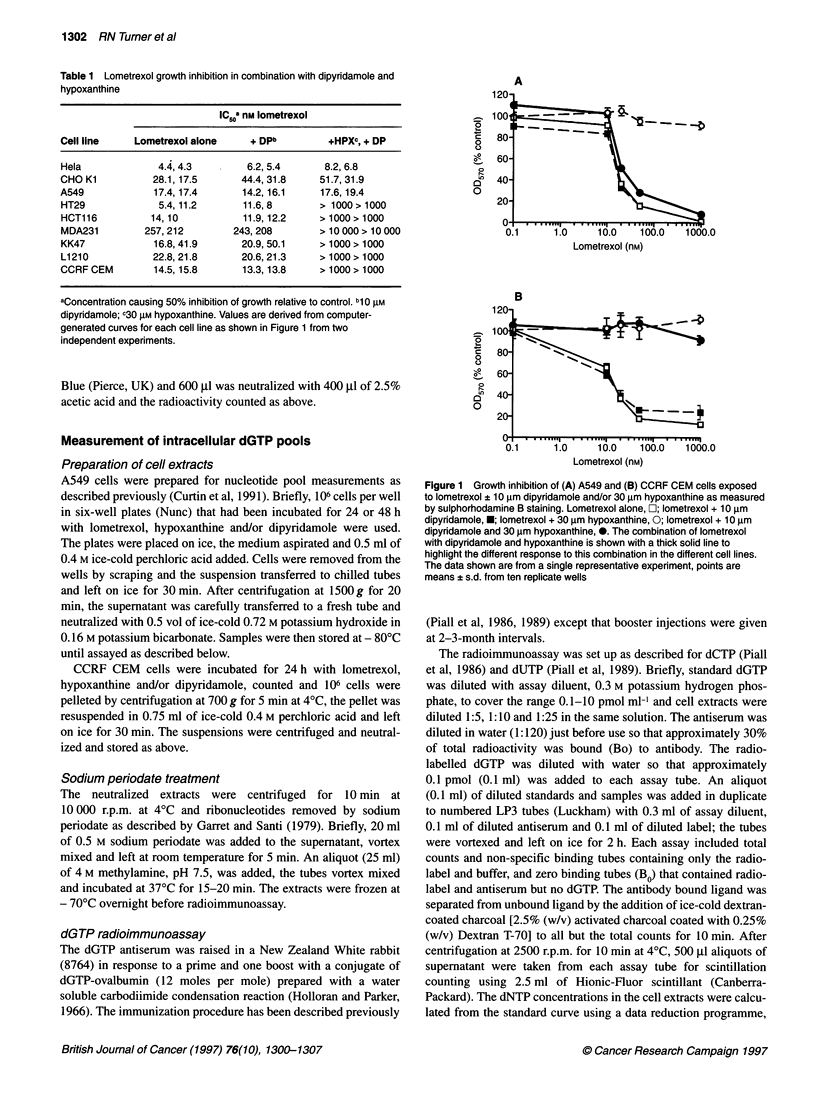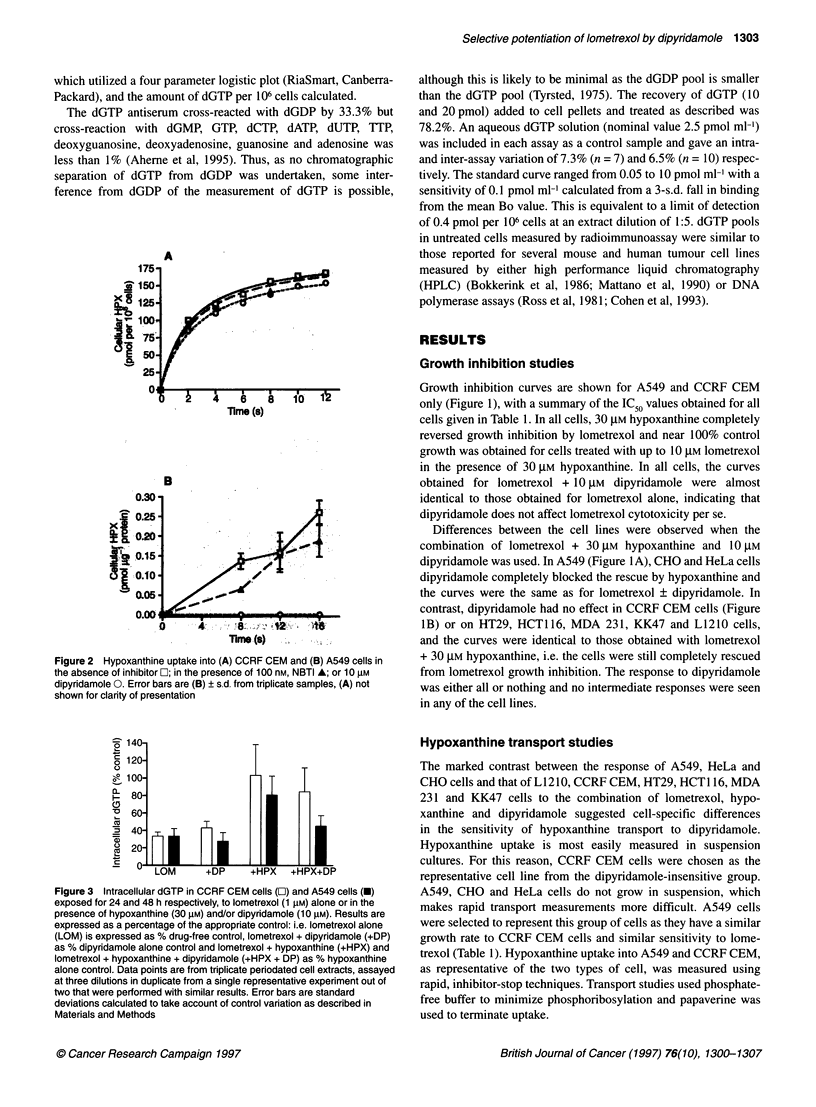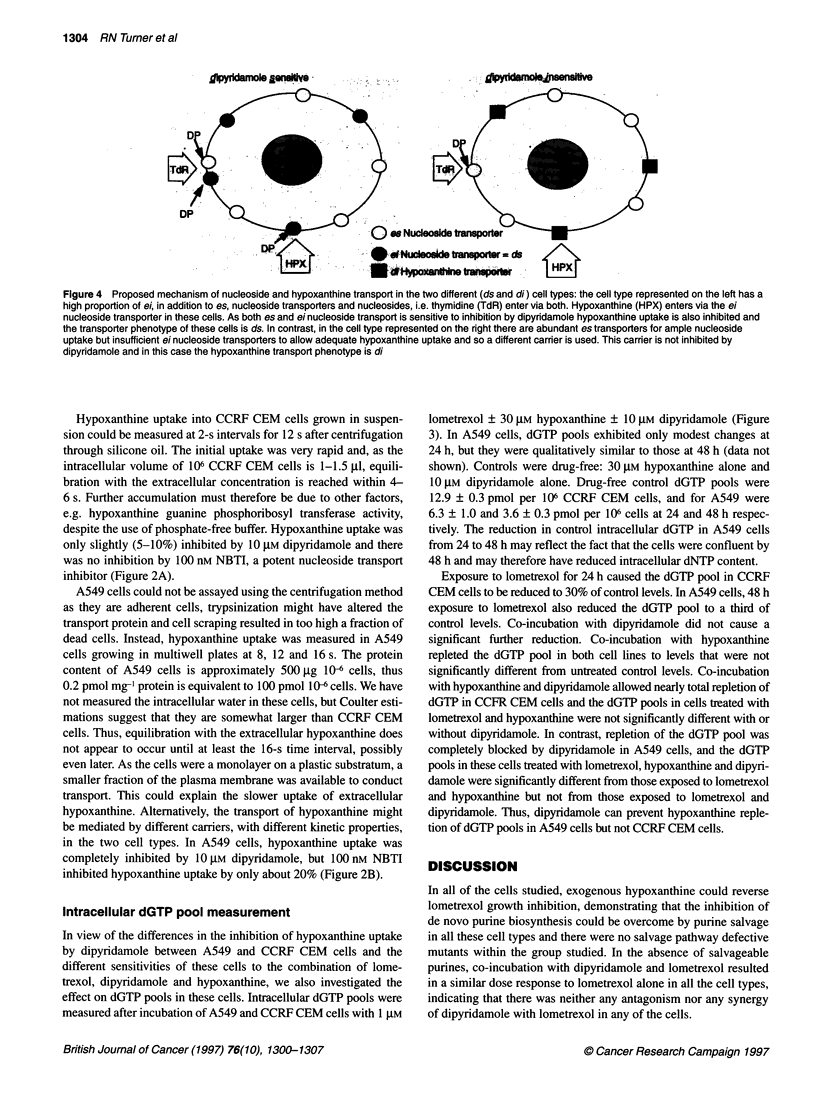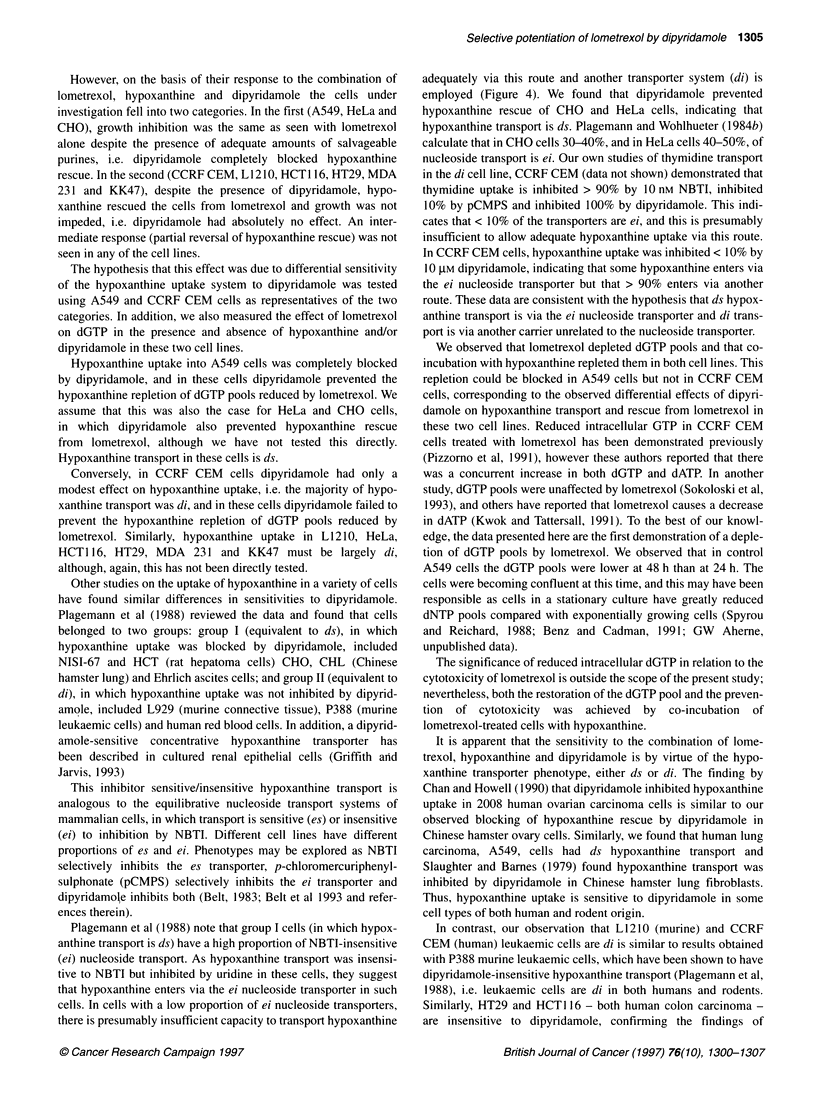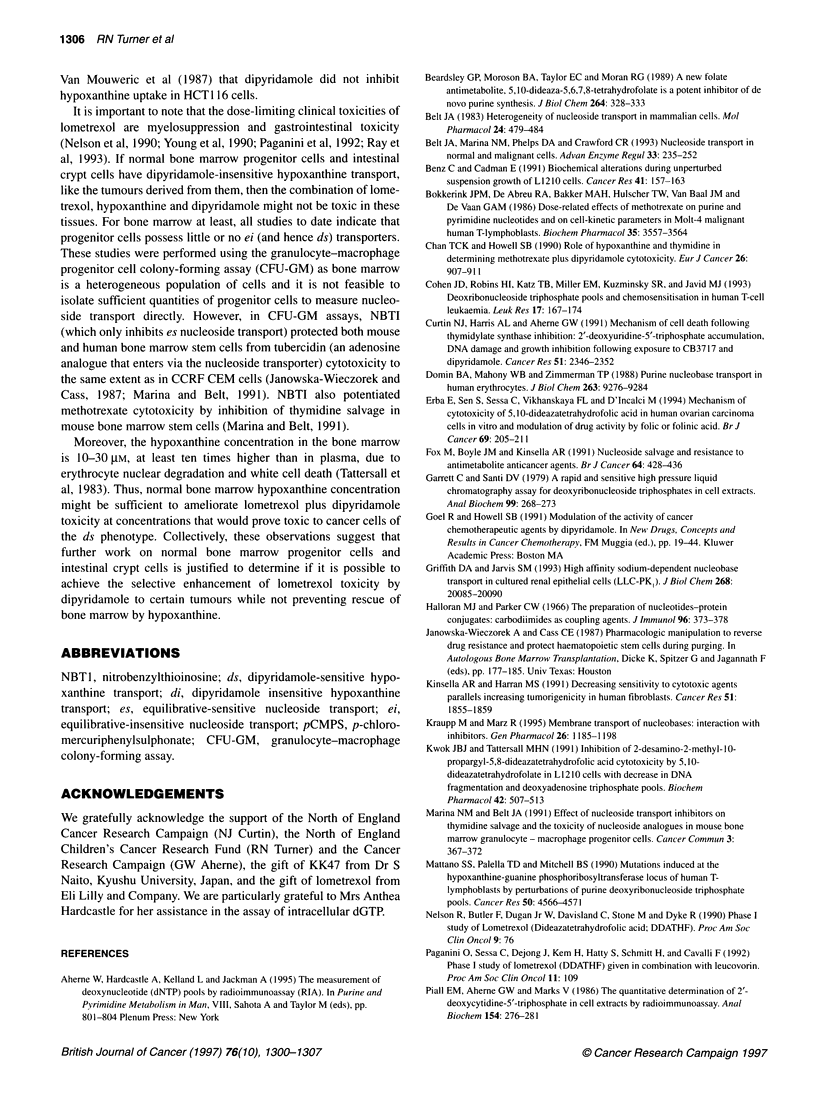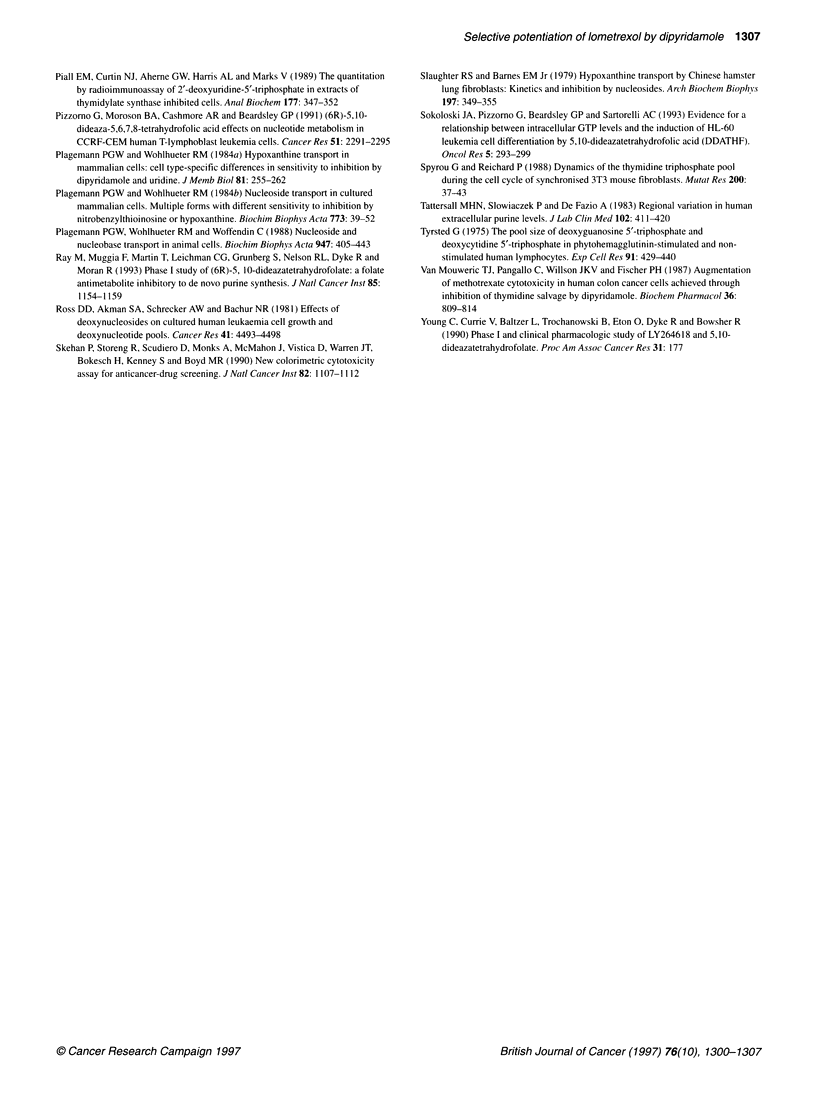# Selective potentiation of lometrexol growth inhibition by dipyridamole through cell-specific inhibition of hypoxanthine salvage.

**DOI:** 10.1038/bjc.1997.552

**Published:** 1997

**Authors:** R. N. Turner, G. W. Aherne, N. J. Curtin

**Affiliations:** Cancer Research Unit, University of Newcastle Upon Tyne, UK.

## Abstract

The novel antifolate lometrexol (5,10-dideazatetrahydrofolate) inhibits de novo purine biosynthesis, and co-incubation with hypoxanthine abolishes its cytotoxicity. The prevention of hypoxanthine rescue from an antipurine antifolate by the nucleoside transport inhibitor dipyridamole was investigated for the first time in nine human and rodent cell lines from seven different tissues of origin. In A549, HeLa and CHO cells, dipyridamole prevented hypoxanthine rescue and so growth was inhibited by the combination of lometrexol, dipyridamole and hypoxanthine, but in HT29, HCT116, KK47, MDA231, CCRF CEM and L1210 cells dipyridamole had no effect and the combination did not inhibit growth. Dipyridamole inhibited hypoxanthine uptake in A549 but not in CCRF CEM cells. Dipyridamole prevented the hypoxanthine-induced repletion of dGTP pools, depleted by lometrexol, in A549 but not in CCRF CEM cells. Thus, the selective growth-inhibitory effect of the combination of lometrexol, dipyridamole and hypoxanthine is apparently due to the dipyridamole sensitivity (ds) or insensitivity (di) of hypoxanthine transport. Both the human and murine leukaemic cells are of the di phenotype. If this reflects the transport phenotype of normal bone marrow it would suggest that the combination of lometrexol, dipyridamole and hypoxanthine might be selectively toxic to certain tumour types and have reduced toxicity to the bone marrow.


British Journal of Cancer (1997) 76(10), 1300–1307
© 1997 Cancer Research Campaign

### Selective potentiation of lometrexol growth inhibition by dipyridamole through cell-specific inhibition of hypoxanthine salvage

**RN Turner[1]*, GW Aherne[2] and NJ Curtin[1]**

[1]Cancer Research Unit, University of Newcastle Upon Tyne, Newcastle upon Tyne, NE2 4HH, UK; [2]CRC Centre for Cancer Therapeutics, Institute of Cancer Research, Sutton, Surrey SM2 5NG, UK

**Summary** The novel antifolate lometrexol (5,10-dideazatetrahydrofolate) inhibits de novo purine biosynthesis, and co-incubation with hypoxanthine abolishes its cytotoxicity. The prevention of hypoxanthine rescue from an antipurine antifolate by the nucleoside transport inhibitor dipyridamole was investigated for the first time in nine human and rodent cell lines from seven different tissues of origin. In A549, HeLa and CHO cells, dipyridamole prevented hypoxanthine rescue and so growth was inhibited by the combination of lometrexol, dipyridamole and hypoxanthine, but in HT29, HCT116, KK47, MDA231, CCRF CEM and L1210 cells dipyridamole had no effect and the combination did not inhibit growth. Dipyridamole inhibited hypoxanthine uptake in A549 but not in CCRF CEM cells. Dipyridamole prevented the hypoxanthine-induced repletion of dGTP pools, depleted by lometrexol, in A549 but not in CCRF CEM cells. Thus, the selective growth-inhibitory effect of the combination of lometrexol, dipyridamole and hypoxanthine is apparently due to the *d*ipyridamole *s*ensitivity (*ds*) or *i*nsensitivity (*di*) of hypoxanthine transport. Both the human and murine leukaemic cells are of the *di* phenotype. If this reflects the transport phenotype of normal bone marrow it would suggest that the combination of lometrexol, dipyridamole and hypoxanthine might be selectively toxic to certain tumour types and have reduced toxicity to the bone marrow.

**Keywords:** lometrexol; dipyridamole; hypoxanthine transport; selective potentiation

Host toxicity and drug resistance are major obstacles to effective and safe chemotherapy, and there is a continuing search for chemotherapeutic combinations that will overcome them. The antifolate lometrexol (5,10-dideaza-5,6,7,8-tetrahydrofolate: DDATHF) represents a novel approach to antifolate chemotherapy as its target for inhibition is glycinamide ribonucleotide transformylase, the first folate-dependent enzyme of de novo purine biosynthesis (Beardsley et al, 1989). Clinically, lometrexol has been associated with some myelosuppression and gastrointestinal toxicity (Nelson et al, 1990; Young et al, 1990; Paganini et al, 1992; Ray et al, 1993) which may be prevented by folic or folinic acid supplementation before and after dosing with lometrexol. This has, however, the potential for a parallel reduction in the anti-tumour activity of lometrexol, although this has not been demonstrated clinically. A means of either selectively increasing the anti-tumour activity or reducing the dose-limiting toxicities, or both, would therefore be desirable.

Resistance to antimetabolite inhibitors of de novo nucleotide biosynthesis may arise as a result of salvage of extracellular nucleosides and nucleobases leading to repletion of intracellular nucleotide pools. Clinically, this may be exacerbated by two factors. First, malignant cells frequently have higher salvage activity than normal cells (Fox et al, 1991; Kinsella and Harran, 1991). Second, release of nucleic acids from dead cells and their breakdown can result in locally high concentrations of salvageable nucleosides and bases in tumours.

The nucleoside transport inhibitor dipyridamole has been used successfully in vitro and to a limited extent in animals and in cancer patients to increase the cytotoxicity of antimetabolites by blocking the uptake of nucleosides for salvage (reviewed by Goel and Howell, 1991). Less is known about the modulation of antimetabolite cytotoxicity via the effect of dipyridamole on nucleobase transport. However, in a review of published data, Plagemann et al (1988) observed that dipyridamole could inhibit hypoxanthine transport in five out of seven rodent cell lines. Furthermore, Chan and Howell (1990) demonstrated the potentiation of methotrexate cytotoxicity in ovarian cancer cells by dipyridamole by inhibition of not only thymidine but also hypoxanthine uptake. Cells treated with lometrexol have reduced ATP and GTP pools; co-incubation with hypoxanthine both repletes these pools (Pizzorno et al, 1991) and abolishes the cytotoxic effect of lometrexol (Erba et al, 1994). Thus, dipyridamole should enhance lometrexol cytotoxicity by the inhibition of hypoxanthine uptake.

We report here an investigation of the efficacy of dipyridamole at preventing hypoxanthine-mediated rescue from lometrexol growth inhibition in a range of human and rodent cell lines (selected to reflect some of the common human malignancies). Hypoxanthine was used at 30 μM in these studies as this represents a physiologically relevant concentration (Tattersall et al, 1983) and dipyridamole was used at 10 μM as this concentration has been shown to inhibit hypoxanthine transport > 90% in other cell lines

*Received 12 March 1997*
*Revised 1 May 1997*
*Accepted 6 May 1997*

*Correspondence to:* NJ Curtin, Cancer Research Unit, University of Newcastle upon Tyne, Medical School, Framlington Place, Newcastle upon Tyne NE2 4HH, UK

**Present address:* South Cleveland General Hospital, Middlesborough, TS4 3BW, UK.

(Plagemann and Wohlhueter, 1984*a*). We believe that this is the first study of the potentiation of an antipurine antifolate by the nucleoside transport inhibitor dipyridamole.

We found that the cells were of two types: those in which dipyridamole could inhibit hypoxanthine rescue and those in which dipyridamole could not prevent hypoxanthine rescue. The uptake of hypoxanthine in two of the cell lines (representing the two types of cell) was measured in the presence and absence of dipyridamole and nitrobenzylthioinosine (NBTI), another nucleoside transport inhibitor. We observed that hypoxanthine uptake was not sensitive to NBTI in either cell line. However, the two cell lines displayed a differential sensitivity to dipyridamole, corresponding to the prevention of hypoxanthine rescue from lometrexol in growth inhibition studies. Furthermore, dipyridamole prevented the hypoxanthine-induced repletion of dGTP pools in the sensitive but not the insensitive cells treated with lometrexol. These data suggest that, for some tumour types, it may be possible to enhance selectively the anti-tumour activity of lometrexol with dipyridamole.

### MATERIALS AND METHODS

### Cell lines

A549 (human lung carcinoma), CCRF CEM (human lymphoblastic leukaemia), CHO K1 (Chinese hamster ovary), HeLa (human cervical adenocarcinoma), HCT116 and HT29, (human colonic carcinoma), KK47 (human transitional cell bladder carcinoma, a gift from Dr S Naito, Kyushu University, Japan), L1210 (mouse lymphocytic leukaemia) and MDA 231 (human breast carcinoma) were all adapted for growth in RPMI-1640 medium (Life Technologies, Paisley, UK) supplemented with 10% dialysed fetal calf serum (Globepharm, Esher, UK). Routine maintenance and all experiments were carried out in medium containing dialysed serum to which a defined concentration of hypoxanthine could be added. All incubations were at 37°C in a humidified atmosphere of 5% carbon dioxide in air.

### Chemicals

All routine chemicals and dipyridamole were obtained from Sigma (Poole, UK), dGTP and dextran T-70 were obtained from Pharmacia (Milton Keynes, UK), [methyl-$^{3}$H]thymidine, deoxy(1′,2′-[$^{3}$H]-guanosine-5′-triphosphate), [$^{3}$H]hypoxanthine and [$^{14}$C]sucrose were obtained from Amersham, UK, and lometrexol was a kind gift from the late G Grindey, Eli Lilly.

### Growth inhibition assays

Drug incubation periods were for 48, 72 or 96 h, depending on the growth rate of the cell under study, to ensure that control cultures had undergone at least three cell doublings during the exposure period.

*Exponentially growing suspension cultures*

L1210 and CCRF CEM cells were seeded at a final density of $10^4$ cells ml$^{-1}$, 0.5 ml per well into 24-well plates (Nunc, Life Technologies) in medium containing hypoxanthine, dipyridamole and lometrexol as indicated in the Results section. After 48 h L1210 cells were counted and after 96 h CCRF CEM cells were counted after fixation with Carnoy's fixative (methanol:acetic acid 3:1). All cell counts were made on a model Z1 Coulter Counter (Coulter Electronics, UK).

*Exponentially growing cultures of adherent cells*

A549, CHO K1, HCT116, HeLa, HT29, KK47 and MDA 231 were seeded into 96-well plates (Nunc) at 1–1.5 × $10^3$ cells per well in 100 µl of medium and left to attach for 8–24 h. The medium was replaced with medium containing the appropriate drug concentrations. Replicate wells were fixed with Carnoy's fixative to estimate the cell density at the start of the drug exposure period. After 72 h (CHO K1) or 96 h (all others) incubation, cells were fixed with Carnoy's fixative, washed, air dried and stained with sulphorhodamine B, as described previously (Skehan et al, 1990). The plates were then read on a Dynatech MR7000 microtitre-plate reader using a 570-nm filter. The optical density was measured relative to an air blank. Data were expressed as % control by the following formula:

$$\text{Per cent control} = (T/C) \times 100 \pm ((T/C) \times 100) \times \sqrt{((c/C)^2 + (t/T)^2)}$$

mean ± standard deviation where $C$ and $c$ are the mean and standard deviation of the control and $T$ and $t$ are the mean and standard deviation of the treated sample respectively.

### Hypoxanthine uptake measurement

Hypoxanthine uptake was measured in exponentially growing CCRF CEM cells by a modification of the 'inhibitor-stop' method of Domin et al (1988). Cells were centrifuged, washed and resuspended in ice-cold 10 mM Hepes–saline pH 7.4 buffer at a density of 2 × $10^7$ cells per ml. Aliquots of cell suspension were incubated with 1% dimethylsulphoxide (DMSO) ± 10 µM dipyridamole or 100 nM NBTI at 37°C for 5 min. An aliquot (100 µl) of this was then carefully layered onto silicone oil (sp.gr. 1.028) (BDH, UK) overlaying 50 µl 3 M potassium hydroxide in each of six 0.4-ml microcentrifuge tubes (BDH). Uptake was initiated by adding 50 µl of 300 µM hypoxanthine containing 25 µCi ml$^{-1}$ [$^{3}$H]hypoxanthine and 2 µCi ml$^{-1}$ [$^{14}$C]sucrose (to correct for extracellular water) to each tube at 1-s intervals (final hypoxanthine concentration = 100 µM, $10^6$ cells per tube). Uptake was terminated by adding 50 µl ice-cold 19 mM papaverine-saline (Kraupp and Marz, 1995) at 1-s intervals in reverse order. The tubes were immediately centrifuged at 14 000 r.p.m. for 1 min and the cells allowed to lyse in the potassium hydroxide for 1 h. The tubes were cut in the oil layer and the bottom portion transferred to scintillation vials, neutralized with 1 ml of 0.25 M acetic acid, 10 ml of scintillant was added and the samples were counted on a Wallac 1410 β-counter.

Hypoxanthine uptake into A549 cells was measured using monolayers 80–90% confluent in 24-well plates (Nunc) by a modification of published methodology (Slaughter and Barnes, 1979). The medium was aspirated from three wells and the wells gently washed three times with Hepes–saline buffer. Cells were then incubated for 5 min in 0.5 ml of the same buffer with 1% DMSO ± 10 µM dipyridamole or 100 µM NBTI at 37°C. Transport was initiated by aspirating the buffer and adding 300 µl buffer containing 100 µM hypoxanthine + 3.3 µCi [$^{3}$H]hypoxanthine and 1 µCi [$^{14}$C]sucrose ml$^{-1}$ [+ 1000-fold excess (1.6 mM) sucrose to minimize non-specific binding] with or without the desired concentration of inhibitor. Uptake was terminated after 8, 12 or 16 s with 0.5 ml of ice-cold 19 mM papaverine followed by immediate aspiration and two further papaverine washes. Cells were then lysed with 1 ml of 0.2 M sodium hydroxide at 60°C for 30 min. Duplicate 100 µl aliquots were taken for protein estimation by Coomassie

© Cancer Research Campaign 1997

**Table 1** Lometrexol growth inhibition in combination with dipyridamole and hypoxanthine

| | $IC_{50}$[a] nM lometrexol | | |
|---|---|---|---|
| **Cell line** | **Lometrexol alone** | **+ DP**[b] | **+HPX**[c]**, + DP** |
| Hela | 4.4, 4.3 | 6.2, 5.4 | 8.2, 6.8 |
| CHO K1 | 28.1, 17.5 | 44.4, 31.8 | 51.7, 31.9 |
| A549 | 17.4, 17.4 | 14.2, 16.1 | 17.6, 19.4 |
| HT29 | 5.4, 11.2 | 11.6, 8 | > 1000 > 1000 |
| HCT116 | 14, 10 | 11.9, 12.2 | > 1000 > 1000 |
| MDA231 | 257, 212 | 243, 208 | > 10 000 > 10 000 |
| KK47 | 16.8, 41.9 | 20.9, 50.1 | > 1000 > 1000 |
| L1210 | 22.8, 21.8 | 20.6, 21.3 | > 1000 > 1000 |
| CCRF CEM | 14.5, 15.8 | 13.3, 13.8 | > 1000 > 1000 |

[a]Concentration causing 50% inhibition of growth relative to control. [b]10 μM dipyridamole; [c]30 μM hypoxanthine. Values are derived from computer-generated curves for each cell line as shown in Figure 1 from two independent experiments.

**Figure 1** Growth inhibition of (**A**) A549 and (**B**) CCRF CEM cells exposed to lometrexol ± 10 μm dipyridamole and/or 30 μm hypoxanthine as measured by sulphorhodamine B staining. Lometrexol alone, □; lometrexol + 10 μm dipyridamole, ■; lometrexol + 30 μm hypoxanthine, ○; lometrexol + 10 μm dipyridamole and 30 μm hypoxanthine, ●. The combination of lometrexol with dipyridamole and hypoxanthine is shown with a thick solid line to highlight the different response to this combination in the different cell lines. The data shown are from a single representative experiment, points are means ± s.d. from ten replicate wells

Blue (Pierce, UK) and 600 μl was neutralized with 400 μl of 2.5% acetic acid and the radioactivity counted as above.

### Measurement of intracellular dGTP pools

### *Preparation of cell extracts*

A549 cells were prepared for nucleotide pool measurements as described previously (Curtin et al, 1991). Briefly, $10^6$ cells per well in six-well plates (Nunc) that had been incubated for 24 or 48 h with lometrexol, hypoxanthine and/or dipyridamole were used. The plates were placed on ice, the medium aspirated and 0.5 ml of 0.4 M ice-cold perchloric acid added. Cells were removed from the wells by scraping and the suspension transferred to chilled tubes and left on ice for 30 min. After centrifugation at 1500 *g* for 20 min, the supernatant was carefully transferred to a fresh tube and neutralized with 0.5 vol of ice-cold 0.72 M potassium hydroxide in 0.16 M potassium bicarbonate. Samples were then stored at – 80°C until assayed as described below.

CCRF CEM cells were incubated for 24 h with lometrexol, hypoxanthine and/or dipyridamole, counted and $10^6$ cells were pelleted by centrifugation at 700 *g* for 5 min at 4°C, the pellet was resuspended in 0.75 ml of ice-cold 0.4 M perchloric acid and left on ice for 30 min. The suspensions were centrifuged and neutralized and stored as above.

### *Sodium periodate treatment*

The neutralized extracts were centrifuged for 10 min at 10 000 r.p.m. at 4°C and ribonucleotides removed by sodium periodate as described by Garret and Santi (1979). Briefly, 20 ml of 0.5 M sodium periodate was added to the supernatant, vortex mixed and left at room temperature for 5 min. An aliquot (25 ml) of 4 M methylamine, pH 7.5, was added, the tubes vortex mixed and incubated at 37°C for 15–20 min. The extracts were frozen at – 70°C overnight before radioimmunoassay.

### *dGTP radioimmunoassay*

The dGTP antiserum was raised in a New Zealand White rabbit (8764) in response to a prime and one boost with a conjugate of dGTP-ovalbumin (12 moles per mole) prepared with a water soluble carbodiimide condensation reaction (Holloran and Parker, 1966). The immunization procedure has been described previously (Piall et al, 1986, 1989) except that booster injections were given at 2–3-month intervals.

The radioimmunoassay was set up as described for dCTP (Piall et al, 1986) and dUTP (Piall et al, 1989). Briefly, standard dGTP was diluted with assay diluent, 0.3 M potassium hydrogen phosphate, to cover the range 0.1–10 pmol $ml^{-1}$ and cell extracts were diluted 1:5, 1:10 and 1:25 in the same solution. The antiserum was diluted in water (1:120) just before use so that approximately 30% of total radioactivity was bound (Bo) to antibody. The radiolabelled dGTP was diluted with water so that approximately 0.1 pmol (0.1 ml) was added to each assay tube. An aliquot (0.1 ml) of diluted standards and samples was added in duplicate to numbered LP3 tubes (Luckham) with 0.3 ml of assay diluent, 0.1 ml of diluted antiserum and 0.1 ml of diluted label; the tubes were vortexed and left on ice for 2 h. Each assay included total counts and non-specific binding tubes containing only the radiolabel and buffer, and zero binding tubes ($B_0$) that contained radiolabel and antiserum but no dGTP. The antibody bound ligand was separated from unbound ligand by the addition of ice-cold dextran-coated charcoal [2.5% (w/v) activated charcoal coated with 0.25% (w/v) Dextran T-70] to all but the total counts for 10 min. After centrifugation at 2500 r.p.m. for 10 min at 4°C, 500 μl aliquots of supernatant were taken from each assay tube for scintillation counting using 2.5 ml of Hionic-Fluor scintillant (Canberra-Packard). The dNTP concentrations in the cell extracts were calculated from the standard curve using a data reduction programme,

© Cancer Research Campaign 1997

which utilized a four parameter logistic plot (RiaSmart, Canberra-Packard), and the amount of dGTP per $10^6$ cells calculated.

The dGTP antiserum cross-reacted with dGDP by 33.3% but cross-reaction with dGMP, GTP, dCTP, dATP, dUTP, TTP, deoxyguanosine, deoxyadenosine, guanosine and adenosine was less than 1% (Aherne et al, 1995). Thus, as no chromatographic separation of dGTP from dGDP was undertaken, some interference from dGDP of the measurement of dGTP is possible, although this is likely to be minimal as the dGDP pool is smaller than the dGTP pool (Tyrsted, 1975). The recovery of dGTP (10 and 20 pmol) added to cell pellets and treated as described was 78.2%. An aqueous dGTP solution (nominal value 2.5 pmol $ml^{-1}$) was included in each assay as a control sample and gave an intra- and inter-assay variation of 7.3% ($n = 7$) and 6.5% ($n = 10$) respectively. The standard curve ranged from 0.05 to 10 pmol $ml^{-1}$ with a sensitivity of 0.1 pmol $ml^{-1}$ calculated from a 3-s.d. fall in binding from the mean Bo value. This is equivalent to a limit of detection of 0.4 pmol per $10^6$ cells at an extract dilution of 1:5. dGTP pools in untreated cells measured by radioimmunoassay were similar to those reported for several mouse and human tumour cell lines measured by either high performance liquid chromatography (HPLC) (Bokkerink et al, 1986; Mattano et al, 1990) or DNA polymerase assays (Ross et al, 1981; Cohen et al, 1993).

**Figure 2** Hypoxanthine uptake into (**A**) CCRF CEM and (**B**) A549 cells in the absence of inhibitor □; in the presence of 100 nM, NBTI ▲; or 10 μM dipyridamole ○. Error bars are (**B**) ± s.d. from triplicate samples, (**A**) not shown for clarity of presentation

**Figure 3** Intracellular dGTP in CCRF CEM cells (□) and A549 cells (■) exposed for 24 and 48 h respectively, to lometrexol (1 μM) alone or in the presence of hypoxanthine (30 μM) and/or dipyridamole (10 μM). Results are expressed as a percentage of the appropriate control: i.e. lometrexol alone (LOM) is expressed as % drug-free control, lometrexol + dipyridamole (+DP) as % dipyridamole alone control and lometrexol + hypoxanthine (+HPX) and lometrexol + hypoxanthine + dipyridamole (+HPX + DP) as % hypoxanthine alone control. Data points are from triplicate periodated cell extracts, assayed at three dilutions in duplicate from a single representative experiment out of two that were performed with similar results. Error bars are standard deviations calculated to take account of control variation as described in Materials and Methods

### RESULTS

### Growth inhibition studies

Growth inhibition curves are shown for A549 and CCRF CEM only (Figure 1), with a summary of the $IC_{50}$ values obtained for all cells given in Table 1. In all cells, 30 μM hypoxanthine completely reversed growth inhibition by lometrexol and near 100% control growth was obtained for cells treated with up to 10 μM lometrexol in the presence of 30 μM hypoxanthine. In all cells, the curves obtained for lometrexol + 10 μM dipyridamole were almost identical to those obtained for lometrexol alone, indicating that dipyridamole does not affect lometrexol cytotoxicity per se.

Differences between the cell lines were observed when the combination of lometrexol + 30 μM hypoxanthine and 10 μM dipyridamole was used. In A549 (Figure 1A), CHO and HeLa cells dipyridamole completely blocked the rescue by hypoxanthine and the curves were the same as for lometrexol ± dipyridamole. In contrast, dipyridamole had no effect in CCRF CEM cells (Figure 1B) or on HT29, HCT116, MDA 231, KK47 and L1210 cells, and the curves were identical to those obtained with lometrexol + 30 μM hypoxanthine, i.e. the cells were still completely rescued from lometrexol growth inhibition. The response to dipyridamole was either all or nothing and no intermediate responses were seen in any of the cell lines.

### Hypoxanthine transport studies

The marked contrast between the response of A549, HeLa and CHO cells and that of L1210, CCRF CEM, HT29, HCT116, MDA 231 and KK47 cells to the combination of lometrexol, hypoxanthine and dipyridamole suggested cell-specific differences in the sensitivity of hypoxanthine transport to dipyridamole. Hypoxanthine uptake is most easily measured in suspension cultures. For this reason, CCRF CEM cells were chosen as the representative cell line from the dipyridamole-insensitive group. A549, CHO and HeLa cells do not grow in suspension, which makes rapid transport measurements more difficult. A549 cells were selected to represent this group of cells as they have a similar growth rate to CCRF CEM cells and similar sensitivity to lometrexol (Table 1). Hypoxanthine uptake into A549 and CCRF CEM, as representative of the two types of cell, was measured using rapid, inhibitor-stop techniques. Transport studies used phosphate-free buffer to minimize phosphoribosylation and papaverine was used to terminate uptake.

**Figure 4** Proposed mechanism of nucleoside and hypoxanthine transport in the two different (*ds* and *di*) cell types: the cell type represented on the left has a high proportion of *ei*, in addition to *es*, nucleoside transporters and nucleosides, i.e. thymidine (TdR) enter via both. Hypoxanthine (HPX) enters via the *ei* nucleoside transporter in these cells. As both *es* and *ei* nucleoside transport is sensitive to inhibition by dipyridamole hypoxanthine uptake is also inhibited and the transporter phenotype of these cells is *ds*. In contrast, in the cell type represented on the right there are abundant *es* transporters for ample nucleoside uptake but insufficient *ei* nucleoside transporters to allow adequate hypoxanthine uptake and so a different carrier is used. This carrier is not inhibited by dipyridamole and in this case the hypoxanthine transport phenotype is *di*

Hypoxanthine uptake into CCRF CEM cells grown in suspension could be measured at 2-s intervals for 12 s after centrifugation through silicone oil. The initial uptake was very rapid and, as the intracellular volume of $10^6$ CCRF CEM cells is 1–1.5 µl, equilibration with the extracellular concentration is reached within 4–6 s. Further accumulation must therefore be due to other factors, e.g. hypoxanthine guanine phosphoribosyl transferase activity, despite the use of phosphate-free buffer. Hypoxanthine uptake was only slightly (5–10%) inhibited by 10 µM dipyridamole and there was no inhibition by 100 nM NBTI, a potent nucleoside transport inhibitor (Figure 2A).

A549 cells could not be assayed using the centrifugation method as they are adherent cells, trypsinization might have altered the transport protein and cell scraping resulted in too high a fraction of dead cells. Instead, hypoxanthine uptake was measured in A549 cells growing in multiwell plates at 8, 12 and 16 s. The protein content of A549 cells is approximately 500 µg $10^{-6}$ cells, thus 0.2 pmol $mg^{-1}$ protein is equivalent to 100 pmol $10^{-6}$ cells. We have not measured the intracellular water in these cells, but Coulter estimations suggest that they are somewhat larger than CCRF CEM cells. Thus, equilibration with the extracellular hypoxanthine does not appear to occur until at least the 16-s time interval, possibly even later. As the cells were a monolayer on a plastic substratum, a smaller fraction of the plasma membrane was available to conduct transport. This could explain the slower uptake of extracellular hypoxanthine. Alternatively, the transport of hypoxanthine might be mediated by different carriers, with different kinetic properties, in the two cell types. In A549 cells, hypoxanthine uptake was completely inhibited by 10 µM dipyridamole, but 100 nM NBTI inhibited hypoxanthine uptake by only about 20% (Figure 2B).

### Intracellular dGTP pool measurement

In view of the differences in the inhibition of hypoxanthine uptake by dipyridamole between A549 and CCRF CEM cells and the different sensitivities of these cells to the combination of lometrexol, dipyridamole and hypoxanthine, we also investigated the effect on dGTP pools in these cells. Intracellular dGTP pools were measured after incubation of A549 and CCRF CEM cells with 1 µM lometrexol ± 30 µM hypoxanthine ± 10 µM dipyridamole (Figure 3). In A549 cells, dGTP pools exhibited only modest changes at 24 h, but they were qualitatively similar to those at 48 h (data not shown). Controls were drug-free: 30 µM hypoxanthine alone and 10 µM dipyridamole alone. Drug-free control dGTP pools were 12.9 ± 0.3 pmol per $10^6$ CCRF CEM cells, and for A549 were 6.3 ± 1.0 and 3.6 ± 0.3 pmol per $10^6$ cells at 24 and 48 h respectively. The reduction in control intracellular dGTP in A549 cells from 24 to 48 h may reflect the fact that the cells were confluent by 48 h and may therefore have reduced intracellular dNTP content.

Exposure to lometrexol for 24 h caused the dGTP pool in CCRF CEM cells to be reduced to 30% of control levels. In A549 cells, 48 h exposure to lometrexol also reduced the dGTP pool to a third of control levels. Co-incubation with dipyridamole did not cause a significant further reduction. Co-incubation with hypoxanthine repleted the dGTP pool in both cell lines to levels that were not significantly different from untreated control levels. Co-incubation with hypoxanthine and dipyridamole allowed nearly total repletion of dGTP in CCFR CEM cells and the dGTP pools in cells treated with lometrexol and hypoxanthine were not significantly different with or without dipyridamole. In contrast, repletion of the dGTP pool was completely blocked by dipyridamole in A549 cells, and the dGTP pools in these cells treated with lometrexol, hypoxanthine and dipyridamole were significantly different from those exposed to lometrexol and hypoxanthine but not from those exposed to lometrexol and dipyridamole. Thus, dipyridamole can prevent hypoxanthine repletion of dGTP pools in A549 cells but not CCRF CEM cells.

### DISCUSSION

In all of the cells studied, exogenous hypoxanthine could reverse lometrexol growth inhibition, demonstrating that the inhibition of de novo purine biosynthesis could be overcome by purine salvage in all these cell types and there were no salvage pathway defective mutants within the group studied. In the absence of salvageable purines, co-incubation with dipyridamole and lometrexol resulted in a similar dose response to lometrexol alone in all the cell types, indicating that there was neither any antagonism nor any synergy of dipyridamole with lometrexol in any of the cells.

© Cancer Research Campaign 1997

However, on the basis of their response to the combination of lometrexol, hypoxanthine and dipyridamole the cells under investigation fell into two categories. In the first (A549, HeLa and CHO), growth inhibition was the same as seen with lometrexol alone despite the presence of adequate amounts of salvageable purines, i.e. dipyridamole completely blocked hypoxanthine rescue. In the second (CCRF CEM, L1210, HCT116, HT29, MDA 231 and KK47), despite the presence of dipyridamole, hypoxanthine rescued the cells from lometrexol and growth was not impeded, i.e. dipyridamole had absolutely no effect. An intermediate response (partial reversal of hypoxanthine rescue) was not seen in any of the cell lines.

The hypothesis that this effect was due to differential sensitivity of the hypoxanthine uptake system to dipyridamole was tested using A549 and CCRF CEM cells as representatives of the two categories. In addition, we also measured the effect of lometrexol on dGTP in the presence and absence of hypoxanthine and/or dipyridamole in these two cell lines.

Hypoxanthine uptake into A549 cells was completely blocked by dipyridamole, and in these cells dipyridamole prevented the hypoxanthine repletion of dGTP pools reduced by lometrexol. We assume that this was also the case for HeLa and CHO cells, in which dipyridamole also prevented hypoxanthine rescue from lometrexol, although we have not tested this directly. Hypoxanthine transport in these cells is *ds*.

Conversely, in CCRF CEM cells dipyridamole had only a modest effect on hypoxanthine uptake, i.e. the majority of hypoxanthine transport was *di*, and in these cells dipyridamole failed to prevent the hypoxanthine repletion of dGTP pools reduced by lometrexol. Similarly, hypoxanthine uptake in L1210, HeLa, HCT116, HT29, MDA 231 and KK47 must be largely *di*, although, again, this has not been directly tested.

Other studies on the uptake of hypoxanthine in a variety of cells have found similar differences in sensitivities to dipyridamole. Plagemann et al (1988) reviewed the data and found that cells belonged to two groups: group I (equivalent to *ds*), in which hypoxanthine uptake was blocked by dipyridamole, included NISI-67 and HCT (rat hepatoma cells) CHO, CHL (Chinese hamster lung) and Ehrlich ascites cells; and group II (equivalent to *di*), in which hypoxanthine uptake was not inhibited by dipyridamole, included L929 (murine connective tissue), P388 (murine leukaemic cells) and human red blood cells. In addition, a dipyridamole-sensitive concentrative hypoxanthine transporter has been described in cultured renal epithelial cells (Griffith and Jarvis, 1993)

This inhibitor sensitive/insensitive hypoxanthine transport is analogous to the equilibrative nucleoside transport systems of mammalian cells, in which transport is sensitive (*es*) or insensitive (*ei*) to inhibition by NBTI. Different cell lines have different proportions of *es* and *ei*. Phenotypes may be explored as NBTI selectively inhibits the *es* transporter, *p*-chloromercuriphenylsulphonate (pCMPS) selectively inhibits the *ei* transporter and dipyridamole inhibits both (Belt, 1983; Belt et al 1993 and references therein).

Plagemann et al (1988) note that group I cells (in which hypoxanthine transport is *ds*) have a high proportion of NBTI-insensitive (*ei*) nucleoside transport. As hypoxanthine transport was insensitive to NBTI but inhibited by uridine in these cells, they suggest that hypoxanthine enters via the *ei* nucleoside transporter in such cells. In cells with a low proportion of *ei* nucleoside transporters, there is presumably insufficient capacity to transport hypoxanthine adequately via this route and another transporter system (*di*) is employed (Figure 4). We found that dipyridamole prevented hypoxanthine rescue of CHO and HeLa cells, indicating that hypoxanthine transport is *ds*. Plagemann and Wohlhueter (1984*b*) calculate that in CHO cells 30–40%, and in HeLa cells 40–50%, of nucleoside transport is *ei*. Our own studies of thymidine transport in the *di* cell line, CCRF CEM (data not shown) demonstrated that thymidine uptake is inhibited > 90% by 10 nM NBTI, inhibited 10% by pCMPS and inhibited 100% by dipyridamole. This indicates that < 10% of the transporters are *ei*, and this is presumably insufficient to allow adequate hypoxanthine uptake via this route. In CCRF CEM cells, hypoxanthine uptake was inhibited < 10% by 10 μM dipyridamole, indicating that some hypoxanthine enters via the *ei* nucleoside transporter but that > 90% enters via another route. These data are consistent with the hypothesis that *ds* hypoxanthine transport is via the *ei* nucleoside transporter and *di* transport is via another carrier unrelated to the nucleoside transporter.

We observed that lometrexol depleted dGTP pools and that co-incubation with hypoxanthine repleted them in both cell lines. This repletion could be blocked in A549 cells but not in CCRF CEM cells, corresponding to the observed differential effects of dipyridamole on hypoxanthine transport and rescue from lometrexol in these two cell lines. Reduced intracellular GTP in CCRF CEM cells treated with lometrexol has been demonstrated previously (Pizzorno et al, 1991), however these authors reported that there was a concurrent increase in both dGTP and dATP. In another study, dGTP pools were unaffected by lometrexol (Sokoloski et al, 1993), and others have reported that lometrexol causes a decrease in dATP (Kwok and Tattersall, 1991). To the best of our knowledge, the data presented here are the first demonstration of a depletion of dGTP pools by lometrexol. We observed that in control A549 cells the dGTP pools were lower at 48 h than at 24 h. The cells were becoming confluent at this time, and this may have been responsible as cells in a stationary culture have greatly reduced dNTP pools compared with exponentially growing cells (Spyrou and Reichard, 1988; Benz and Cadman, 1991; GW Aherne, unpublished data).

The significance of reduced intracellular dGTP in relation to the cytotoxicity of lometrexol is outside the scope of the present study; nevertheless, both the restoration of the dGTP pool and the prevention of cytotoxicity was achieved by co-incubation of lometrexol-treated cells with hypoxanthine.

It is apparent that the sensitivity to the combination of lometrexol, hypoxanthine and dipyridamole is by virtue of the hypoxanthine transporter phenotype, either *ds* or *di*. The finding by Chan and Howell (1990) that dipyridamole inhibited hypoxanthine uptake in 2008 human ovarian carcinoma cells is similar to our observed blocking of hypoxanthine rescue by dipyridamole in Chinese hamster ovary cells. Similarly, we found that human lung carcinoma, A549, cells had *ds* hypoxanthine transport and Slaughter and Barnes (1979) found hypoxanthine transport was inhibited by dipyridamole in Chinese hamster lung fibroblasts. Thus, hypoxanthine uptake is sensitive to dipyridamole in some cell types of both human and rodent origin.

In contrast, our observation that L1210 (murine) and CCRF CEM (human) leukaemic cells are *di* is similar to results obtained with P388 murine leukaemic cells, which have been shown to have dipyridamole-insensitive hypoxanthine transport (Plagemann et al, 1988), i.e. leukaemic cells are *di* in both humans and rodents. Similarly, HT29 and HCT116 – both human colon carcinoma – are insensitive to dipyridamole, confirming the findings of

Van Mouweric et al (1987) that dipyridamole did not inhibit hypoxanthine uptake in HCT116 cells.

It is important to note that the dose-limiting clinical toxicities of lometrexol are myelosuppression and gastrointestinal toxicity (Nelson et al, 1990; Young et al, 1990; Paganini et al, 1992; Ray et al, 1993). If normal bone marrow progenitor cells and intestinal crypt cells have dipyridamole-insensitive hypoxanthine transport, like the tumours derived from them, then the combination of lometrexol, hypoxanthine and dipyridamole might not be toxic in these tissues. For bone marrow at least, all studies to date indicate that progenitor cells possess little or no *ei* (and hence *ds*) transporters. These studies were performed using the granulocyte–macrophage progenitor cell colony-forming assay (CFU-GM) as bone marrow is a heterogeneous population of cells and it is not feasible to isolate sufficient quantities of progenitor cells to measure nucleoside transport directly. However, in CFU-GM assays, NBTI (which only inhibits *es* nucleoside transport) protected both mouse and human bone marrow stem cells from tubercidin (an adenosine analogue that enters via the nucleoside transporter) cytotoxicity to the same extent as in CCRF CEM cells (Janowska-Wieczorek and Cass, 1987; Marina and Belt, 1991). NBTI also potentiated methotrexate cytotoxicity by inhibition of thymidine salvage in mouse bone marrow stem cells (Marina and Belt, 1991).

Moreover, the hypoxanthine concentration in the bone marrow is 10–30 μM, at least ten times higher than in plasma, due to erythrocyte nuclear degradation and white cell death (Tattersall et al, 1983). Thus, normal bone marrow hypoxanthine concentration might be sufficient to ameliorate lometrexol plus dipyridamole toxicity at concentrations that would prove toxic to cancer cells of the *ds* phenotype. Collectively, these observations suggest that further work on normal bone marrow progenitor cells and intestinal crypt cells is justified to determine if it is possible to achieve the selective enhancement of lometrexol toxicity by dipyridamole to certain tumours while not preventing rescue of bone marrow by hypoxanthine.

### ABBREVIATIONS

NBT1, nitrobenzylthioinosine; *ds*, dipyridamole-sensitive hypoxanthine transport; *di*, dipyridamole insensitive hypoxanthine transport; *es*, equilibrative-sensitive nucleoside transport; *ei*, equilibrative-insensitive nucleoside transport; *p*CMPS, *p*-chloromercuriphenylsulphonate; CFU-GM, granulocyte–macrophage colony-forming assay.

### ACKNOWLEDGEMENTS

We gratefully acknowledge the support of the North of England Cancer Research Campaign (NJ Curtin), the North of England Children's Cancer Research Fund (RN Turner) and the Cancer Research Campaign (GW Aherne), the gift of KK47 from Dr S Naito, Kyushu University, Japan, and the gift of lometrexol from Eli Lilly and Company. We are particularly grateful to Mrs Anthea Hardcastle for her assistance in the assay of intracellular dGTP.

### REFERENCES

Aherne W, Hardcastle A, Kelland L and Jackman A (1995) The measurement of deoxynucleotide (dNTP) pools by radioimmunoassay (RIA). In *Purine and Pyrimidine Metabolism in Man*, VIII, Sahota A and Taylor M (eds), pp. 801–804 Plenum Press: New York

Beardsley GP, Moroson BA, Taylor EC and Moran RG (1989) A new folate antimetabolite, 5,10-dideaza-5,6,7,8-tetrahydrofolate is a potent inhibitor of de novo purine synthesis. *J Biol Chem* **264**: 328–333

Belt JA (1983) Heterogeneity of nucleoside transport in mammalian cells. *Mol Pharmacol* **24**: 479–484

Belt JA, Marina NM, Phelps DA and Crawford CR (1993) Nucleoside transport in normal and malignant cells. *Advan Enzyme Regul* **33**: 235–252

Benz C and Cadman E (1991) Biochemical alterations during unperturbed suspension growth of L1210 cells. *Cancer Res* **41**: 157–163

Bokkerink JPM, De Abreu RA, Bakker MAH, Hulscher TW, Van Baal JM and De Vaan GAM (1986) Dose-related effects of methotrexate on purine and pyrimidine nucleotides and on cell-kinetic parameters in Molt-4 malignant human T-lymphoblasts. *Biochem Pharmacol* **35**: 3557–3564

Chan TCK and Howell SB (1990) Role of hypoxanthine and thymidine in determining methotrexate plus dipyridamole cytotoxicity. *Eur J Cancer* **26**: 907–911

Cohen JD, Robins HI, Katz TB, Miller EM, Kuzminsky SR, and Javid MJ (1993) Deoxribonucleoside triphosphate pools and chemosensitisation in human T-cell leukaemia. *Leuk Res* **17**: 167–174

Curtin NJ, Harris AL and Aherne GW (1991) Mechanism of cell death following thymidylate synthase inhibition: 2′-deoxyuridine-5′-triphosphate accumulation, DNA damage and growth inhibition following exposure to CB3717 and dipyridamole. *Cancer Res* **51**: 2346–2352

Domin BA, Mahony WB and Zimmerman TP (1988) Purine nucleobase transport in human erythrocytes. *J Biol Chem* **263**: 9276–9284

Erba E, Sen S, Sessa C, Vikhanskaya FL and D'Incalci M (1994) Mechanism of cytotoxicity of 5,10-dideazatetrahydrofolic acid in human ovarian carcinoma cells in vitro and modulation of drug activity by folic or folinic acid. *Br J Cancer* **69**: 205–211

Fox M, Boyle JM and Kinsella AR (1991) Nucleoside salvage and resistance to antimetabolite anticancer agents. *Br J Cancer* **64**: 428–436

Garrett C and Santi DV (1979) A rapid and sensitive high pressure liquid chromatography assay for deoxyribonucleoside triphosphates in cell extracts. *Anal Biochem* **99**: 268–273

Goel R and Howell SB (1991) Modulation of the activity of cancer chemotherapeutic agents by dipyridamole. In *New Drugs, Concepts and Results in Cancer Chemotherapy*, FM Muggia (ed.), pp. 19–44. Kluwer Academic Press: Boston MA

Griffith DA and Jarvis SM (1993) High affinity sodium-dependent nucleobase transport in cultured renal epithelial cells (LLC-$PK_1$). *J Biol Chem* **268**: 20085–20090

Halloran MJ and Parker CW (1966) The preparation of nucleotides–protein conjugates: carbodiimides as coupling agents. *J Immunol* **96**: 373–378

Janowska-Wieczorek A and Cass CE (1987) Pharmacologic manipulation to reverse drug resistance and protect haematopoietic stem cells during purging. In *Autologous Bone Marrow Transplantation*, Dicke K, Spitzer G and Jagannath F (eds), pp. 177–185. Univ Texas: Houston

Kinsella AR and Harran MS (1991) Decreasing sensitivity to cytotoxic agents parallels increasing tumorigenicity in human fibroblasts. *Cancer Res* **51**: 1855–1859

Kraupp M and Marz R (1995) Membrane transport of nucleobases: interaction with inhibitors. *Gen Pharmacol* **26**: 1185–1198

Kwok JBJ and Tattersall MHN (1991) Inhibition of 2-desamino-2-methyl-10-propargyl-5,8-dideazatetrahydrofolic acid cytotoxicity by 5,10-dideazatetrahydrofolate in L1210 cells with decrease in DNA fragmentation and deoxyadenosine triphosphate pools. *Biochem Pharmacol* **42**: 507–513

Marina NM and Belt JA (1991) Effect of nucleoside transport inhibitors on thymidine salvage and the toxicity of nucleoside analogues in mouse bone marrow granulocyte – macrophage progenitor cells. *Cancer Commun* **3**: 367–372

Mattano SS, Palella TD and Mitchell BS (1990) Mutations induced at the hypoxanthine-guanine phosphoribosyltransferase locus of human T-lymphoblasts by perturbations of purine deoxyribonucleoside triphosphate pools. *Cancer Res* **50**: 4566–4571

Nelson R, Butler F, Dugan Jr W, Davisland C, Stone M and Dyke R (1990) Phase I study of Lometrexol (Dideazatetrahydrofolic acid; DDATHF). *Proc Am Soc Clin Oncol* **9**: 76

Paganini O, Sessa C, Dejong J, Kem H, Hatty S, Schmitt H, and Cavalli F (1992) Phase I study of lometrexol (DDATHF) given in combination with leucovorin. *Proc Am Soc Clin Oncol* **11**: 109

Piall EM, Aherne GW and Marks V (1986) The quantitative determination of 2′-deoxycytidine-5′-triphosphate in cell extracts by radioimmunoassay. *Anal Biochem* **154**: 276–281

© Cancer Research Campaign 1997

Piall EM, Curtin NJ, Aherne GW, Harris AL and Marks V (1989) The quantitation by radioimmunoassay of 2′-deoxyuridine-5′-triphosphate in extracts of thymidylate synthase inhibited cells. *Anal Biochem* **177**: 347–352

Pizzorno G, Moroson BA, Cashmore AR and Beardsley GP (1991) (6R)-5,10-dideaza-5,6,7,8-tetrahydrofolic acid effects on nucleotide metabolism in CCRF-CEM human T-lymphoblast leukemia cells. *Cancer Res* **51**: 2291–2295

Plagemann PGW and Wohlhueter RM (1984*a*) Hypoxanthine transport in mammalian cells: cell type-specific differences in sensitivity to inhibition by dipyridamole and uridine. *J Memb Biol* **81**: 255–262

Plagemann PGW and Wohlhueter RM (1984*b*) Nucleoside transport in cultured mammalian cells. Multiple forms with different sensitivity to inhibition by nitrobenzylthioinosine or hypoxanthine. *Biochim Biophys Acta* **773**: 39–52

Plagemann PGW, Wohlhueter RM and Woffendin C (1988) Nucleoside and nucleobase transport in animal cells. *Biochim Biophys Acta* **947**: 405–443

Ray M, Muggia F, Martin T, Leichman CG, Grunberg S, Nelson RL, Dyke R and Moran R (1993) Phase I study of (6R)-5, 10-dideazatetrahydrofolate: a folate antimetabolite inhibitory to de novo purine synthesis. *J Natl Cancer Inst* **85**: 1154–1159

Ross DD, Akman SA, Schrecker AW and Bachur NR (1981) Effects of deoxynucleosides on cultured human leukaemia cell growth and deoxynucleotide pools. *Cancer Res* **41**: 4493–4498

Skehan P, Storeng R, Scudiero D, Monks A, McMahon J, Vistica D, Warren JT, Bokesch H, Kenney S and Boyd MR (1990) New colorimetric cytotoxicity assay for anticancer-drug screening. *J Natl Cancer Inst* **82**: 1107–1112

Slaughter RS and Barnes EM Jr (1979) Hypoxanthine transport by Chinese hamster lung fibroblasts: Kinetics and inhibition by nucleosides. *Arch Biochem Biophys* **197**: 349–355

Sokoloski JA, Pizzorno G, Beardsley GP and Sartorelli AC (1993) Evidence for a relationship between intracellular GTP levels and the induction of HL-60 leukemia cell differentiation by 5,10-dideazatetrahydrofolic acid (DDATHF). *Oncol Res* **5**: 293–299

Spyrou G and Reichard P (1988) Dynamics of the thymidine triphosphate pool during the cell cycle of synchronised 3T3 mouse fibroblasts. *Mutat Res* **200**: 37–43

Tattersall MHN, Slowiaczek P and De Fazio A (1983) Regional variation in human extracellular purine levels. *J Lab Clin Med* **102**: 411–420

Tyrsted G (1975) The pool size of deoxyguanosine 5′-triphosphate and deoxycytidine 5′-triphosphate in phytohemagglutinin-stimulated and non-stimulated human lymphocytes. *Exp Cell Res* **91**: 429–440

Van Mouweric TJ, Pangallo C, Willson JKV and Fischer PH (1987) Augmentation of methotrexate cytotoxicity in human colon cancer cells achieved through inhibition of thymidine salvage by dipyridamole. *Biochem Pharmacol* **36**: 809–814

Young C, Currie V, Baltzer L, Trochanowski B, Eton O, Dyke R and Bowsher R (1990) Phase I and clinical pharmacologic study of LY264618 and 5,10-dideazatetrahydrofolate. *Proc Am Assoc Cancer Res* **31**: 177

© Cancer Research Campaign 1997